# Serum uric acid/creatinine ratio and 1-year stroke recurrence in patient with acute ischemic stroke and abnormal renal function: results from the Xi'an stroke registry study of China

**DOI:** 10.3389/fneur.2025.1496791

**Published:** 2025-02-04

**Authors:** Zhongzhong Liu, Dandan Zhang, Lingxia Zeng, Weiyan Guo, Qingli Lu, Zhen Lei, Yunlong Hao, Pei Liu, Tong Liu, Linna Peng, Qiaoqiao Chang, Mi Zhang, Xuemei Lin, Fang Wang, Songdi Wu

**Affiliations:** ^1^Department of Neurology, Xi'an No. 1 Hospital, The First Affiliated Hospital of Northwest University, Xi'an, China; ^2^Xi'an Key Laboratory for Innovation and Translation of Neuroimmunological Diseases, Xi'an, China; ^3^Department of Epidemiology and Biostatistics, School of Public Health of Xi'an Jiaotong University Health Science Center, Xi'an, China; ^4^College of Life Science, Northwest University, Xi'an, China; ^5^School of Medicine, Xizang Minzu University, Xianyang, Shaanxi, China

**Keywords:** ischemic stroke, renal function, stroke recurrence, outcomes ischemic stroke, outcomes

## Abstract

**Background:**

The relationship between abnormal renal function and serum uric acid levels in patients with acute ischemic stroke (AIS) remains insufficiently explored. Although uric acid is associated with cardiovascular and cerebrovascular risk, the specific link between normalized serum uric acid (SUA/SCr) and stroke recurrence in patients with impaired renal function has not been well studied. This study aims to fill this gap by investigating the association between SUA/SCr and 1-year stroke recurrence in patients with AIS and abnormal renal function.

**Methods:**

This study utilized the ratio of serum uric acid (SUA) to serum creatinine (SCr) to represent SUA levels normalized for renal function. Abnormal Renal function was defined by the estimated glomerular filtration rate (eGFR) < 90 mL/min/1.73 m^2^. Multivariable Cox regression, curve fitting, and stratified analyses were employed to assess the relationship between SUA/SCr and 1-year stroke recurrence in patients with AIS and abnormal renal function, considering SUA/SCr as both a continuous variable and in quartiles (Q1–Q4).

**Results:**

Of 1,932 enrolled patients (65.3% male; mean age 66.7 ± 11.3 years), each unit of increase in SUA/SCr was associated with a 17% decrease in 1-year stroke recurrence (HR = 0.83, 95% CI 0.73 to 0.96, *P* = 0.009). Compared to Q1, the Q2 and Q4 groups showed significantly reduced risk in 1-year stroke recurrence (Q2: HR = 0.46, 95% CI 0.27 to 0.79, *P* = 0.005; Q4: HR = 0.47, 95% CI 0.27 to 0.81, *P* = 0.007), with a significant trend across all quartiles (*P* = 0.01 for trend tests). Curve fitting revealed a negative but non-linear correlation. Subgroup analyses showed that in patients with eGFR < 60 ml/min/1.73 m^2^, Q4 had significantly lower 1-year stroke recurrence risk than Q1 (HR = 0.19, 95% CI 0.04 to 0.86, *P* = 0.031).

**Conclusion:**

Low SUA/SCr independently predicts 1-year stroke recurrence in patients with AIS and abnormal renal function, particularly in those with eGFR < 60 mL/min/1.73 m^2^.

## 1 Introduction

Among hospitalized patients with stroke in China, about 83.3% suffer from acute ischemic stroke (AIS) (1), with an annual recurrence rate of about 9.6%−17.7% (2, 3). Chronic kidney disease (CKD) is prevalent among stroke patients, with decreased renal function observed in 20%−35% of AIS cases (4). The presence of abnormal renal function significantly increases the risk of stroke recurrence in patients with AIS (5, 6). Accurate assessment of the risk factors for stroke recurrence in patients with AIS and abnormal renal function is of great significance for the secondary prevention of ischemic stroke and for taking measures to reduce the risk of recurrence.

Serum uric acid (SUA) is the final metabolite of purine catabolism (7). Previous studies have produced conflicting results regarding the relationship between SUA levels at admission and acute stroke outcomes, with findings varying from positive outcomes (8–10) to insignificant association (11, 12) and negative outcomes (13, 14). In addition, some studies have found a U-shaped association between excessively low or high SUA levels and worse stroke outcomes (15). As SUA is mainly excreted by the kidneys, variations in renal clearance may contribute to the inconsistent results of previous studies (16). Studies have demonstrated that elevated SUA levels are associated with a higher risk of in-hospital mortality in patients with ischemic stroke and kidney disease, whereas this association is not observed in patients with normal renal function. The results of these studies suggest that the impact of kidney function should be considered when evaluating the association of SUA with stroke outcomes (17).

Serum creatinine (SCr) is an important marker for identifying minor alterations in glomerular filtration rate (GFR) and is widely recognized as a dependable biomarker for early detection of chronic kidney disease (CKD) (18). Given that renal function typically influences the renal clearance of SUA, the SUA to SCr ratio (SUA/SCr) has emerged as a novel marker that normalizes SUA to renal function, thereby providing a more accurate reflection of the net SUA level (19–21). Prior research has identified a correlation between the SUA/SCr ratio and conditions such as non-alcoholic fatty liver disease, metabolic syndrome, and impaired islet function (19–21). Nonetheless, the association between the SUA/SCr ratio and stroke recurrence remains underexplored, with limited studies addressing this, particularly in AIS with abnormal renal function, for whom no reports have been published to date. This study sought to explore the association between the SUA/SCr ratio and 1-year stroke recurrence in patients with AIS and abnormal renal function, utilizing data from the Xi'an Stroke Registry Study database. The goal is to offer a scientific foundation for the prevention and treatment of 1-year stroke recurrence in this patient population.

## 2 Materials and methods

### 2.1 Study design and study population

**Figure 1:**
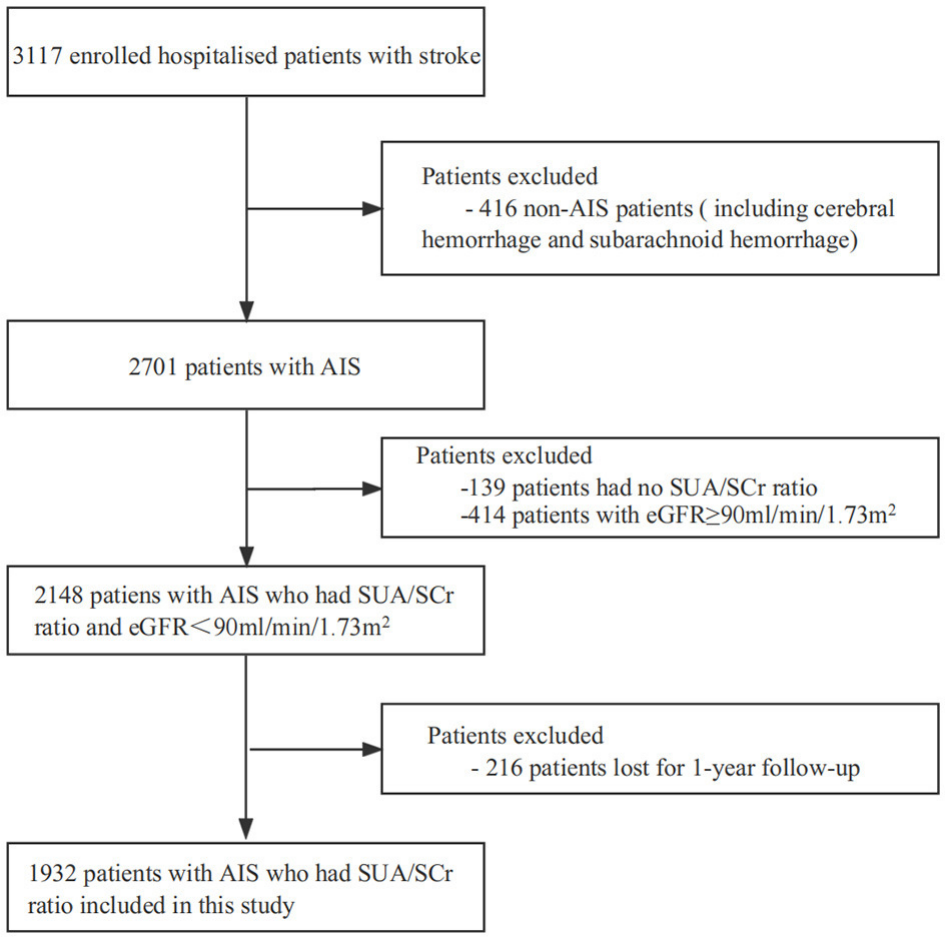
Screening flow charts for enrolled patients. AIS, acute ischemic stroke; SUA, Serum Uric Acid; SCr, Serum Creatinine; eGFR; estimated glomerular filtration rate.

This was a prospective, multicenter, observational cohort study. The study population consisted of patients with stroke admitted to four grade A tertiary hospitals in Xi'an, China, between January and December 2015. At the initial stage of the study, 3,117 patients with stroke underwent a comprehensive medical examination, with follow-up conducted at 1, 3, 6, and 12 months after onset of symptoms. The inclusion criteria were as follows: a clinical diagnosis of AIS, with imaging findings in line with the diagnostic criteria outlined by the American Heart Association/American Stroke Association and confirmed through brain computed tomography or cranial magnetic resonance imaging (22). The patients were between the ages of 18 and 97 years and had experienced symptom onset within 7 days. The exclusion criteria were as follows: patients with cerebral or subarachnoid hemorrhage (*n* = 416), patients with loss of SUA/SCr value (*n* = 139), patients with eGFR ≥ 90 ml/min/1.73 m^2^ (*n* = 414), and patients lost to follow-up (*n* = 216). Ultimately, 1,932 patients with AIS were included in the study. The detailed screening process and study flowchart are presented in Figure 1. Consistent diagnostic criteria were applied across all participating hospitals.

### 2.2 Standard protocol approval and patient consent

This study adhered to the principles outlined in the Declaration of Helsinki. Ethical approval was obtained from the Academic Committee of Xi'an No. 1 Hospital and the ethics committees of all participating hospitals (Approval No. 2014 (5); Registration Number: ChiCTR-EOC-17012190). Written or verbal informed consent was obtained from each patient prior to participation.

### 2.3 Baseline data collection

This multicenter observational cohort study utilized data from the Xi'an Stroke Registry. Baseline information, including sociodemographic details, medical history, admission assessments, and key laboratory results, was collected (Supplementary Table 1). The criteria for medical history, risk factors, and definitions were aligned with those used in the Chinese National Stroke Registry (CNSR) Study (23). SUA/SCr was evaluated as both a continuous and categorical variable (quartiles, Q1–Q4). The quartile ranges for SUA/SCr were as follows, from lowest to highest: Q1: < 3.091, Q2: 3.091–3.895, Q3: 3.896–4.773, Q4: ≥4.774.

### 2.4 Measurements and outcomes

In this study, AIS refers to a type of disease caused by impaired blood supply to brain tissue due to various reasons, leading to ischemic and hypoxic necrosis of brain tissue and ultimately resulting in brain dysfunction. This primarily includes acute cerebral infarction and transient ischemic attack, with diagnostic criteria based on the World Health Organization's standards (24). The SUA/SCr measurements were performed by experienced nurses using blood samples collected on the morning following patient admission. These samples were subsequently sent to the laboratory for analysis. All technicians involved in testing across the hospitals received specialized training, and the instruments used were uniformly calibrated and of identical models. Fasting venous blood samples were obtained the day after admission following overnight fasting. Smoking was defined as the consumption of at least one cigarette per day for a minimum of 6 months prior to stroke onset, while smoking cessation was classified as meeting the smoking criteria previously but refraining from smoking for at least 6 months before the stroke. Alcohol consumption was defined as consuming at least one drink per week, with a standard drink equating to 45 ml of liquor, 360 ml of beer, or 120 ml of wine. Pneumonia was identified within the 1^st^ week post-stroke in patients not requiring mechanical ventilation, diagnosed based on a chest radiograph, clinical symptoms like fever, purulent sputum, and cough, alongside abnormal laboratory findings such as altered white blood cell counts (25). Abnormal Renal function was defined by the estimated glomerular filtration rate (eGFR) < 90 mL/min/1.73 m^2^ (26). The eGFR was determined using the CKD-EPI creatinine equation with a correction factor of 1.1 for the Asian population (27, 28). In this study, the the endpoint event of stroke recurrence refers to the occurrence of a new stroke event (including cerebral infarction, cerebral hemorrhage, and subarachnoid hemorrhage) during the 1-year follow-up period. A committee of four to five stroke experts from each hospital confirmed the endpoint events.

### 2.5 Follow-up

All patients underwent routine follow-up at 1, 3, 6, and 12 months following the AIS diagnosis. Trained study coordinators carried out follow-ups with all enrolled patients either through phone interviews or in-person visits, ensuring that the deviation in follow-up timing did not exceed 5 days and precisely documenting the dates of any stroke recurrence. Patients who withdrew from the study or could not be reached after three daily attempts over five consecutive working days were classified as lost to follow-up.

### 2.6 Statistical analyses

Continuous variables with normal distribution were expressed as mean ± standard deviation, while categorical variables were reported as frequencies and percentages. Non-normally distributed variables were presented as medians with interquartile ranges. Group comparisons were performed using one-way ANOVA, Chi-square test, or non-parametric tests depending on data distribution. Fisher's exact test was applied when expected frequencies were < 10. Multivariable Cox proportional hazards models were used to assess the association between SUA/SCr and 1-year stroke recurrence. The Kaplan-Meier (K-M) method and log-rank test were used to analyze the 1-year cumulative stroke recurrence rate stratified by SUA/SCr quartile groups. Covariates were included in the adjusted model if they altered the SUA/SCr estimates for 1-year stroke recurrence by more than 10% or were significantly related to stroke recurrence at 1 year. Subgroup interactions were evaluated using a likelihood ratio test. Statistical significance was defined as a two-tailed *P-*value < 0.05. All analyses were conducted using Free Statistics software version 1.7 (http://www.clinicalscientists.cn, Free Clinical Medical Technology, Inc., Beijing, China) and R software version 3.3.2 (http://www.R-project.org, The R Foundation).

## 3 Results

### 3.1 Baseline characteristics

After 1 year of follow-up, 216 patients were lost to follow-up, resulting in a final cohort of 1,932 patients. A comparison of clinical characteristics between the followed and lost-to-follow-up groups revealed statistically significant differences only in education level, smoking history, systolic blood pressure (SBP) on admission, and atrial fibrillation, with no significant differences observed in other variables (Supplementary Table 1). This suggests that the key characteristics of patients lost to follow-up were similar to those retained, indicating that the analyzed cohort is representative of the initially enrolled population.

The study included 1,932 patients, comprising 1,261 men and 671 women, with a mean age of 66.7 ± 11.3 years. The average SUA/SCr ratio was 4.0 ± 1.5. Baseline demographic, clinical, and biochemical characteristics across SUA/SCr quartiles (Q1–Q4) were compared (Table 1). There were significant differences in age, presence of co-morbidities such as diabetes mellitus and pneumonia, National Institutes of Health Stroke Scale (NIHSS) score, levels of blood parameters such as triglycerides, fasting plasma glucose (FPG), alanine aminotransferase, homocysteine, aspartate aminotransferase, blood urea nitrogen, serum uric acid, serum creatinine, and white blood cell count, eGFR, and international normalized ratio (INR) among different SUA/SCr values (*P* < 0.05). No statistically significant differences were observed across SUA/SCr quartiles in terms of sex, type of medical insurance, education level, smoking history, alcohol consumption, co-morbidities such as hypertension, atrial fibrillation, and previous stroke, body mass index (BMI), SBP and diastolic blood pressure on admission (DBP), heart rate, or blood parameters including total cholesterol, high-density lipoprotein (HDL) cholesterol, low-density lipoprotein (LDL) cholesterol, glycated hemoglobin, alkaline phosphatase, and platelet count.

**Table 1:** Baseline and biochemical characteristics by SUA/SCr quartiles (Q1-Q4) in patients with AIS and abnormal renal function.

**Variables**	**Overall (*n =* 1,932)**	**SUA/SCr quartiles**				***P*-value**
		**Q1 (*n =* 483)**	**Q2 (*n =* 483)**	**Q3 (*n =* 483)**	**Q4 (*n =* 483)**	
Age (years)	66.7 ± 11.3	67.9 ± 10.4	68.1 ± 10.8	66.3 ± 11.6	64.5 ± 12.0	< 0.001
Sex, *n* (%)						0.346
Male	1,261(65.3)	310(64.2)	328(67.9)	320(66.3)	303(62.7)	
Female	671(34.7)	173(35.8)	155(32.1)	163(33.7)	180(37.3)	
Medical insurance type, *n* (%)						0.064
Urban employees' medical insurance	937(48.5)	212(43.9)	267(55.3)	234(48.4)	224(46.4)	
New type rural cooperative medical system	756(39.1)	206(42.7)	164(34)	187(38.7)	199(41.2)	
Commercial insurance	7(0.4)	3(0.6)	1(0.2)	2(0.4)	1(0.2)	
Out-of-pocket medical	232(12.0)	62(12.8)	51(10.6)	60(12.4)	59(12.2)	
Educational level, *n* (%)						0.55
Elementary or below	932(48.2)	231(47.8)	232(48)	246(50.9)	223(46.2)	
Middle school	369(19.1)	103(21.3)	86(17.8)	84(17.4)	96(19.9)	
High school or above	631(32.7)	149(30.8)	165(34.2)	153(31.7)	164(34)	
Smoking, *n* (%)						0.406
Never smoking	1,058(54.8)	283(58.6)	260(53.8)	252(52.2)	263(54.5)	
Smoking cessation	406(21.0)	91(18.8)	112(23.2)	103(21.3)	100(20.7)	
Current smoking	468(24.2)	109(22.6)	111(23)	128(26.5)	120(24.8)	
Alcohol consumption, *n* (%)						0.114
No	1,478(76.5)	387(80.1)	372(77)	358(74.1)	361(74.7)	
Yes	454(23.5)	96(19.9)	111(23)	125(25.9)	122(25.3)	
Hypertension, n (%)						0.879
No	544(28.2)	134(27.7)	143(29.6)	134(27.7)	133(27.5)	
Yes	1,388(71.8)	349(72.3)	340(70.4)	349(72.3)	350(72.5)	
Diabetes mellitus, *n* (%)						0.026
No	1,458(75.5)	342(70.8)	367(76)	382(79.1)	367(76)	
Yes	474(24.5)	141(29.2)	116(24)	101(20.9)	116(24)	
Atrial fibrillation, *n* (%)						0.818
No	1,779(92.1)	443(91.7)	441(91.3)	448(92.8)	447(92.5)	
Yes	153(7.9)	40(8.3)	42(8.7)	35(7.2)	36(7.5)	
Prior stroke, *n* (%)						0.315
No	1,355(70.1)	324(67.1)	337(69.8)	346(71.6)	348(72)	
Yes	577(29.9)	159(32.9)	146(30.2)	137(28.4)	135(28)	
Pneumonia*, n* (%)						0.027
No	1,816(94.0)	441(91.3)	462(95.7)	455(94.2)	458(94.8)	
Yes	116(6.0)	42(8.7)	21(4.3)	28(5.8)	25(5.2)	
BMI (kg/m^2^)	23.7 ± 3.4	23.6 ± 3.7	23.6 ± 3.1	23.6 ± 3.2	24.1 ± 3.5	0.058
SBP on admission (mmHg)	146.6 ± 21.9	147.0 ± 22.7	146.1 ± 21.4	146.4 ± 21.7	146.9 ± 21.9	0.887
DBP on admission (mmHg)	85.5 ± 12.5	85.9 ± 13.3	85.2 ± 11.8	84.7 ± 11.6	86.3 ± 13.2	0.189
HR (beats/min)	75.0 ± 10.9	75.4 ± 11.5	74.5 ± 10.6	74.8 ± 11.2	75.1 ± 10.3	0.589
Admission NIHSS score, (IQR)	4.0(2.0,6.0)	4.0(2.0,7.0)	4.0(1.0,6.0)	4.0(2.0,6.0)	4.0(2.0,6.0)	0.027
Total cholesterol (mmol/L)	4.4 ± 1.1	4.4 ± 1.0	4.3 ± 1.1	4.4 ± 1.0	4.4 ± 1.2	0.731
Triglycerides (mmol/L)	1.7 ± 1.4	1.5 ± 1.2	1.6 ± 1.1	1.7 ± 1.4	1.9 ± 1.7	< 0.001
HDL cholesterol (mmol/L)	1.1 ± 0.3	1.1 ± 0.3	1.1 ± 0.3	1.1 ± 0.3	1.1 ± 0.3	0.434
LDL cholesterol (mmol/L)	2.6 ± 0.8	2.6 ± 0.8	2.6 ± 0.8	2.6 ± 0.8	2.6 ± 0.8	0.711
Glycated hemoglobin (%)	6.4 ± 1.6	6.6 ± 1.9	6.5 ± 1.7	6.2 ± 1.4	6.3 ± 1.5	0.068
FPG (mmol/L)	6.0 ± 2.5	6.5 ± 2.9	5.9 ± 2.5	5.8 ± 2.1	6.0 ± 2.2	< 0.001
Alanine aminotransferase (U/L)	18.6(14.0,27.0)	18.1(13.0,25.0)	18.0(13.0,25.0)	18.0(14.0,27.0)	21.0(14.9,30.0)	< 0.001
Aspartate Aminotransferase (U/L)	21.0(17.0,28.0)	21.1(17.0,29.0)	20.6(17.0,26.0)	21.0(17.0,27.0)	22.0(18.0,29.0)	0.016
Alkaline phosphatase (U/L)	79.8 ± 28.7	81.0 ± 30.0	78.0 ± 24.5	81.0 ± 26.9	79.3 ± 32.6	0.306
Homocysteine (μmol/L)	18.0(12.4,27.5)	19.6(14.2,30.8)	18.3(12.6,27.5)	17.2(11.9,26.2)	16.7(11.3,24.7)	< 0.001
eGFR (mL/min/1.73m^2^)	72.5 ± 13.3	68.0 ± 17.4	74.0 ± 11.3	75.7 ± 9.7	72.4 ± 12.2	< 0.001
Serum creatinine (μmol/L)	76.9 ± 38.7	95.3 ± 66.6	78.5 ± 20.6	71.1 ± 16.2	62.6 ± 17.3	< 0.001
Blood urea nitrogen (mmol/L)	5.2 ± 2.0	5.7 ± 2.4	5.3 ± 2.0	5.1 ± 1.6	4.9 ± 1.7	< 0.001
Serum uric acid (μmol/L)	288.2 ± 98.3	210.0 ± 92.2	275.5 ± 73.1	306.9 ± 70.1	360.4 ± 89.8	< 0.001
SUA/SCr	4.0 ± 1.5	2.3 ± 0.8	3.5 ± 0.2	4.3 ± 0.2	5.9 ± 1.3	< 0.001
White blood cell ( × 10^9^/L)	7.0 ± 2.6	7.3 ± 3.1	6.9 ± 2.5	6.9 ± 2.4	7.1 ± 2.4	0.043
Platelet count ( × 10^9^/L)	187.4 ± 60.3	183.2 ± 63.1	189.8 ± 63.1	188.1 ± 56.5	188.6 ± 58.1	0.35
INR	1.0 ± 0.2	1.1 ± 0.3	1.0 ± 0.2	1.0 ± 0.1	1.0 ± 0.2	0.008

AIS, Acute ischemic stroke; BMI, body mass index; SBP, Systolic Blood Pressure; DBP, Diastolic Blood Pressure; HR, Heart Rate; NIHSS, National Institutes of Health Stroke Scale; HLD, High-Density Lipoprotein; LDL, Low-density Lipoprotein; FPG, Fasting Venous Plasma Glucose; eGFR, estimated Glomerular Filtration Rate; INR, international normalized ratio; IQR, Interquartile Range; Q, quartiles. Q1: < 3.091, Q2: 3.091–3.895, Q3: 3.896–4.773, Q4: ≥4.774.

### 3.2 Association between SUA/SCr and 1-year stroke recurrence in patients with AIS and abnormal renal function

**Figure 2:**
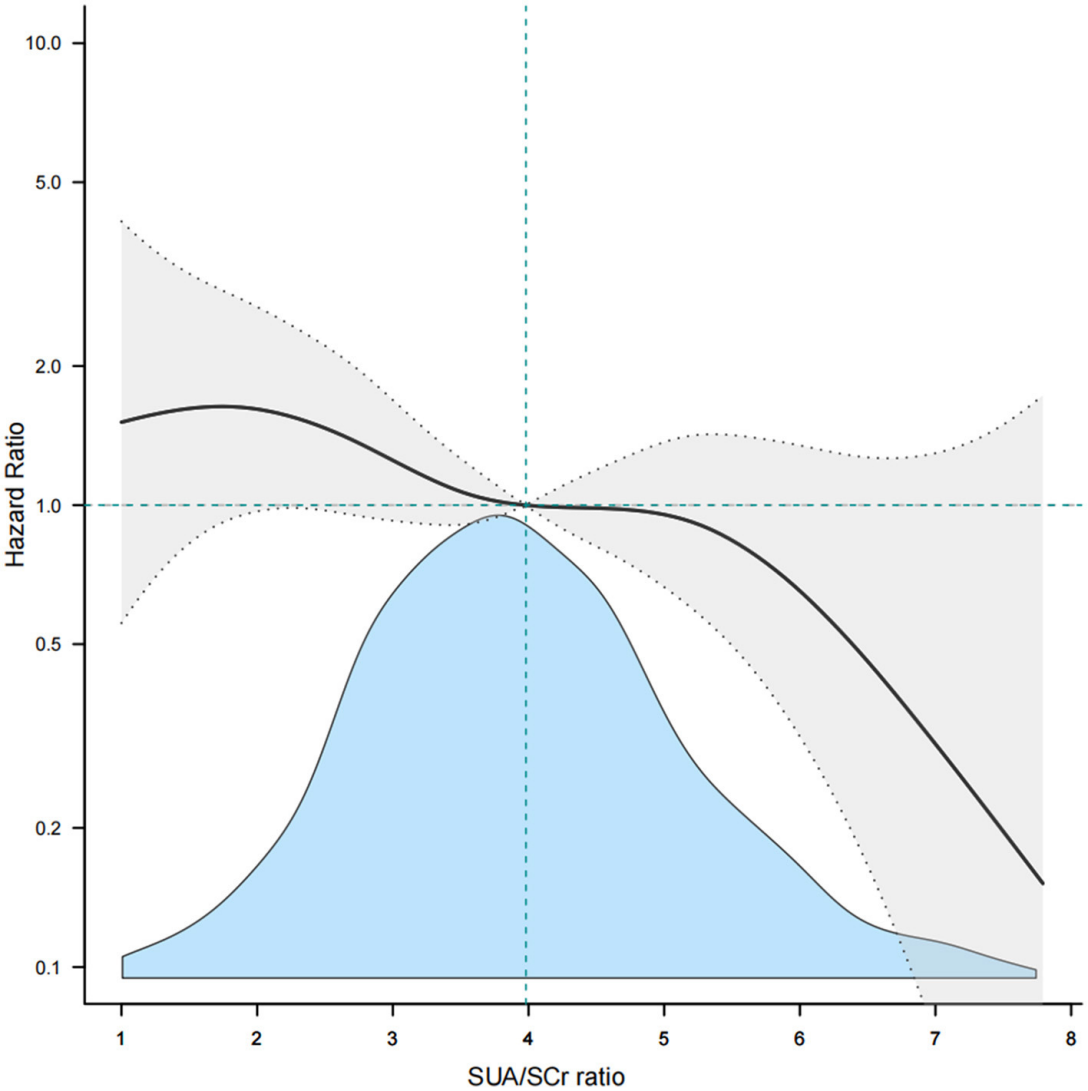
Smooth curve fitting of SUA/SCr to 1-year stroke recurrence. SUA, Serum Uric Acid; SCr, Serum Creatinine.

**Figure 3:**
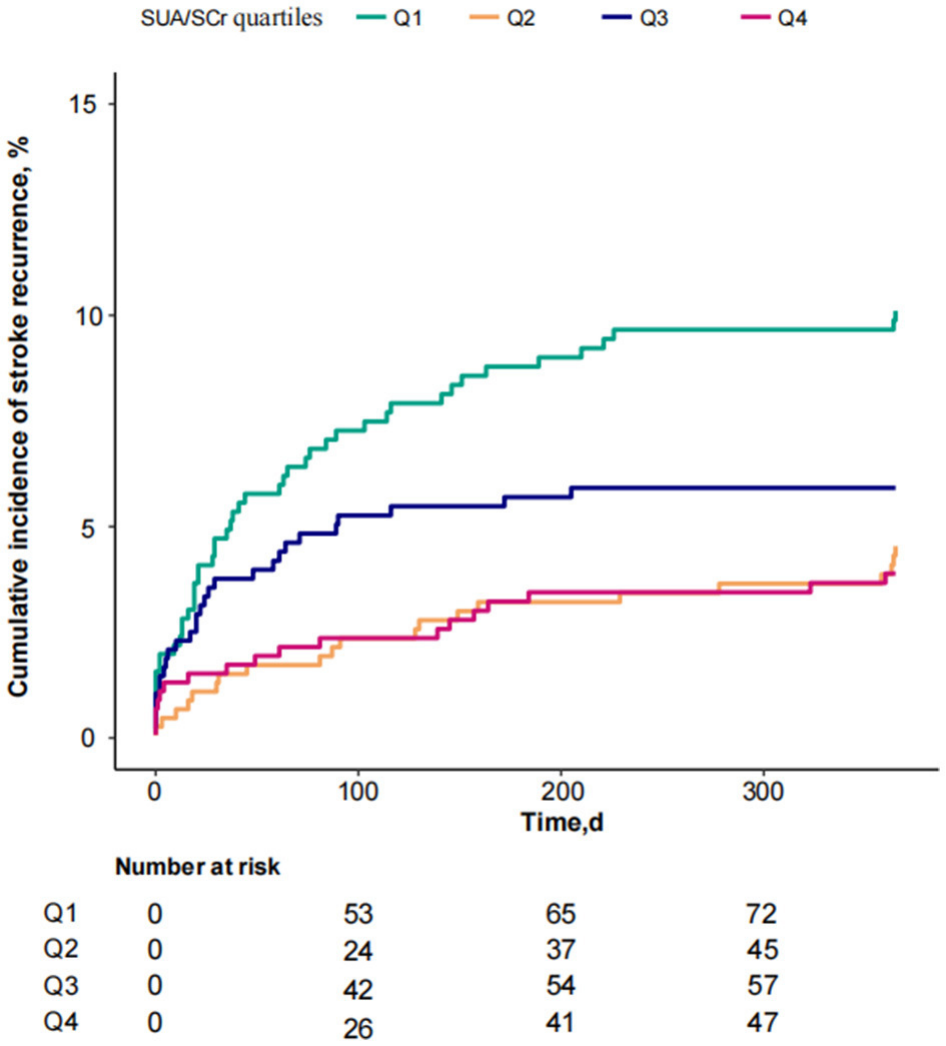
KM curve analysis of 1-year stroke recurrence in patients with AIS and abnormal renal function stratified by SUA/SCr quartiles.

Considering SUA/SCr as a continuous variable, multivariable Cox regression analysis, adjusted for confounders, revealed a 17% decrease in 1-year stroke recurrence risk for each 1-unit increase in SUA/SCr (HR = 0.83, 95% CI 0.73 to 0.96, *P* = 0.009) (Table 2). When SUA/SCr was categorized into quartiles, with Q1 as the reference, the risk of 1-year stroke recurrence was 54% lower in Q2 (HR = 0.46, 95% CI 0.27 to 0.79, *P* = 0.005) and 53% lower in Q4 (HR = 0.47, 95% CI 0.27 to 0.81, *P* = 0.007) compared to Q1, with no significant difference observed between Q3 and Q1. Trend analysis indicated a significant decreasing trend in stroke recurrence risk across the quartiles from Q1 to Q4 (*P* = 0.01) (Table 2). Further curve-fitting analysis suggested a negative but non-linear association between SUA/SCr and 1-year stroke recurrence in patients withAIS and abnormal renal function (Figure 2). KM curve analysis also indicated that, compared to the Q1 group, the 1-year stroke recurrence risk in patients with AISand abnormal renal function was significantly lower in the Q2 and Q4 (Figure 3).

**Table 2:** Multivariable Cox analyses of the SUA/SCr and 1-year stroke recurrence in patients with AIS and abnormal renal function.

**Outcomes**	**Overall, *n***	**Event, *n* (%)**	**Crude model HR (95%CI)**	***P*-value**	**Adjusted model HR (95%CI)**	***P*-value**
**Stroke recurrence**						
SUA/SCr, per 1 unit increase	1,932	113 (5.8)	0.78 (0.68~0.89)	< 0.001	0.83 (0.73~0.96)	0.009
**SUA/SCr quartiles**						
Q1	483	47 (9.7)	1 (Ref)		1 (Ref)	
Q2	483	21 (4.3)	0.43 (0.26~0.72)	0.001	0.46 (0.27~0.79)	0.005
Q3	483	27 (5.6)	0.56 (0.35~0.91)	0.018	0.63 (0.39~1.03)	0.066
Q4	483	18 (3.7)	0.37 (0.21~0.63)	< 0.001	0.47 (0.27~0.81)	0.007
Trend test				0.001		0.01

Crude model adjust none; Adjusted model adjust for age, sex, smoking, alcohol consumption, prior stroke, NIHSS score at admission, pneumonia, BMI, alkaline phosphatase, WBC, hypertension, diabetes mellitus, and atrial fibrillation. HR, hazard ratio; CI, confidence interval; Q, quartiles. Q1: < 3.091, Q2: 3.091–3.895, Q3: 3.896–4.773, Q4: ≥4.774.

### 3.3 Association of SUA/SCr with 1-year stroke recurrence stratified according to preservation of renal function

Based on previous reports on renal dysfunction (29, 30), eGFR ≥ 60 ml/min/1.73 m^2^ was defined as preserved renal function. Therefore, patients with AIS and abnormal renal function were stratified into two groups: eGFR < 60 ml/min/1.73 m^2^ and ≥60 ml/min/1.73 m^2^. In patients with eGFR < 60 ml/min/1.73 m^2^, the risk of 1-year stroke recurrence in the Q4 group significantly decreased compared with the Q1 group after adjusting for potential confounders (HR = 0.19, 95% CI 0.04 to 0.86, *P* = 0.031). The trend of stroke recurrence significantly differed between groups Q1 and Q4 both in the crude and adjusted models (*P* = 0.034 and *P* = 0.013 for trend tests, respectively) (Table 3). Among patients with eGFR ≥ 60 ml/min/1.73 m^2^, the risk of 1-year stroke recurrence was reduced significantly in both the Q2 and Q4 groups compared with the Q1 group in a crude model (Q2: HR = 0.43, 95% CI 0.24 to 0.79, *P* = 0.007; Q4: HR = 0.44, 95% CI 0.24 to 0.81, *P* = 0.008). The trend of stroke recurrence was significantly different between Q1 and Q4 (*P* = 0.023). After adjustment for potential confounding factors, compared with the Q1 group, the risk of 1-year stroke recurrence in group Q2 was significantly reduced (HR = 0.48, 95% CI 0.25 to 0.9, *P* = 0.023), while the risk of 1-year stroke recurrence in group Q4 was not significantly different (HR = 0.67, 95% CI 0.36 to 1.26, *P* = 0.216). There was also no statistically significant difference in the trend of recurrence between Q1 and Q4 (*P* = 0.345).

**Table 3:** SUA/SCr quartiles and 1-year stroke recurrence were analyzed separately stratified according to eGFR value.

**Outcomes**	**Overall, *n***	**Event, *n* (%)**	**Crude model HR (95%CI)**	***P*-value**	**Adjusted model HR (95%CI)**	***P*-value**
**eGFR**						
**< 60 mL/min/1.73m** ^2^						
Q1	128	17 (13.3)	1(Ref)		1(Ref)	
Q2	61	5 (8.2)	0.61 (0.22~1.65)	0.327	0.47 (0.17~1.35)	0.163
Q3	33	3 (9.1)	0.68 (0.2~2.32)	0.538	0.43 (0.11~1.61)	0.207
Q4	64	2 (3.1)	0.22 (0.05~0.96)	0.044	0.19 (0.04~0.86)	0.031
Trend test				0.034		0.013
≥**60 mL/min/1.73m**^2^						
Q1	355	30 (8.5)	1(Ref)		1(Ref)	
Q2	422	16 (3.8)	0.43 (0.24~0.79)	0.007	0.48 (0.25~0.9)	0.023
Q3	450	24 (5.3)	0.63 (0.37~1.08)	0.09	0.75 (0.43~1.31)	0.31
Q4	419	16 (3.8)	0.44 (0.24~0.81)	0.008	0.67 (0.36~1.26)	0.216
Trend test				0.023		0.345

Crude model adjust none; Adjusted model adjust for age, sex, smoking, alcohol consumption, prior stroke, NIHSS score at admission, pneumonia, BMI, alkaline phosphatase, WBC, hypertension, diabetes mellitus, and atrial fibrillation. HR, hazard ratio; CI, confidence interval; Q, quartiles. Q1: < 3.091, Q2: 3.091–3.895, Q3: 3.896–4.773, Q4: ≥ 4.774.

### 3.4 Subgroup analyses

**Figure 4:**
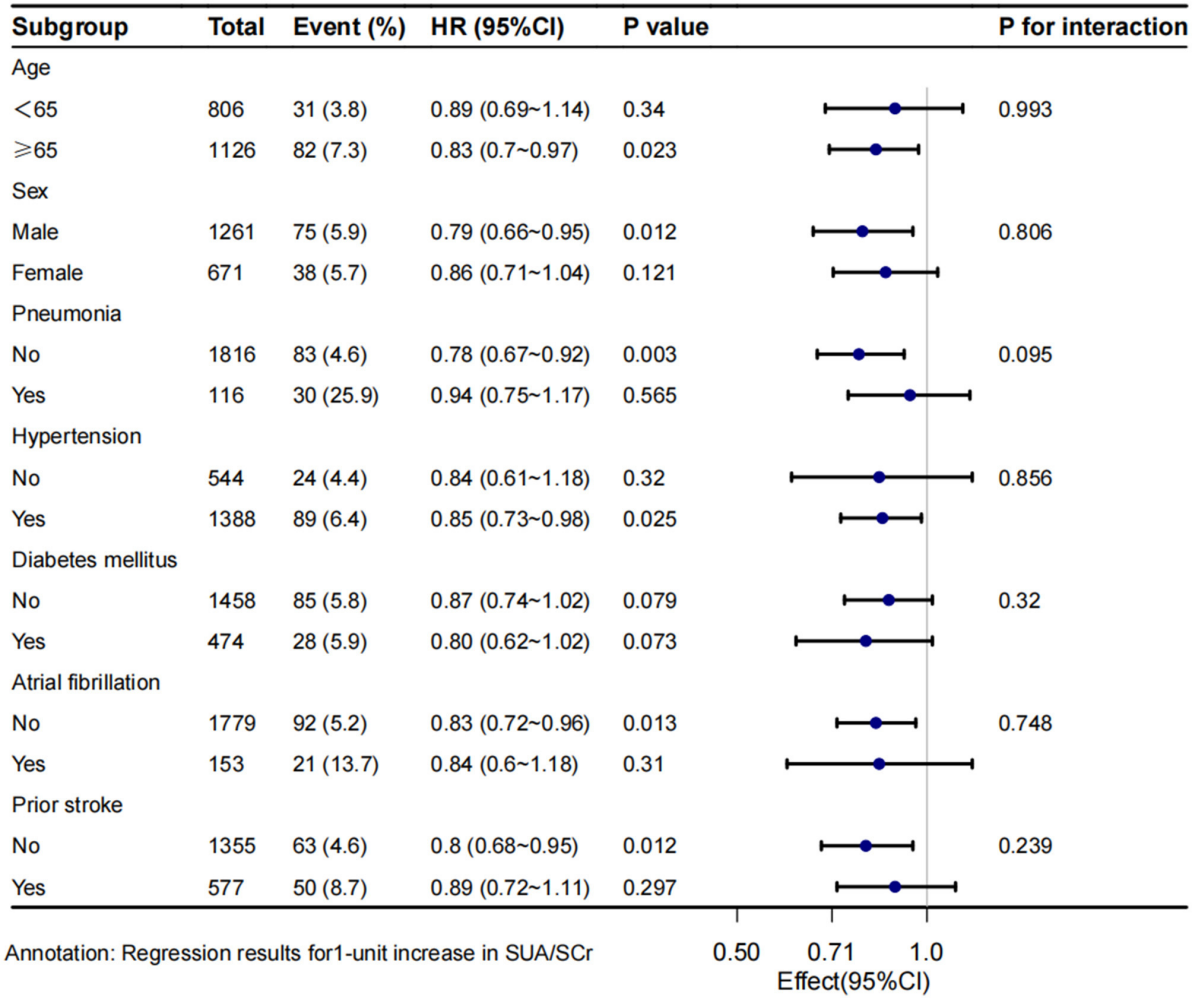
Subgroup analysis of SUA/SCr and 1-year stroke recurrence in patients with AIS and abnormal renal function. Crude model adjust none; Adjusted model adjust for sex, smoking, alcohol consumption, prior stroke, NIHSS score at admission, BMI, alkaline phosphatase, WBC. HR, hazard ratio; CI, confidence interval.

Stratified and interaction analyses were conducted to assess the consistency of the association between SUA/SCr and 1-year stroke recurrence across different subgroups (Figure 4). The results revealed that there was no significant interaction between age, sex, and the presence of co-morbidities such as pneumonia, hypertension, diabetes mellitus, atrial fibrillation, and prior stroke,. However, stratified analysis still found a lower risk of 1-year stroke recurrence in patients with age ≥65 years (HR = 0.83, 95% CI 0.7 to 0.97, *P* = 0.023), male patients (HR = 0.79, 95% CI 0.66 to 0.95, *P* = 0.012), those without pneumonia (HR = 0.78, 95% CI 0.67 to 0.92, *P* = 0.003), those with hypertension (HR = 0.85, 95% CI 0.73 to 0.98, *P* = 0.025), those without atrial fibrillation (HR = 0.83, 95% CI 0.72 to 0.96, *P* = 0.013), and those without prior stroke (HR = 0.8, 95% CI 0.68 to 0.95, *P* = 0.012).

## 4 Discussion

In this multicenter prospective cohort study, we identified low SUA/SCr as an independent risk factor for 1-year stroke recurrence, especially in patients with eGFR < 60 mL/min/1.73 m^2^, revealing a non-linear negative relationship between the two variables. Subgroup analyses showed that in patients with eGFR < 60 ml/min/1.73 m^2^, Q4 had significantly lower 1-year stroke recurrence risk than Q1. These results underscores the need for clinicians to closely monitor SUA/SCr levels in AIS patients with renal impairment.

Previous studies have demonstrated that a lower SUA/SCr ratio has been linked to poorer functional outcomes at 3 months and 1 year in AIS patients, and SUA/SCr is also positively correlated with the risk of metabolically unhealthy phenotypes (31, 32). Notably, recent studies have shown that SUA/SCr is positively associated with the risk of stroke recurrence in young ischemic stroke patients (33). This is not consistent with our findings from this multicenter study, where we found that lower SUA/SCr levels were associated with 1-year stroke recurrence. Variances in research outcomes can be ascribed to discrepancies in patient populations, number of patients, research approaches, and regions. These findings, all related to stroke, suggest that abnormal SUA/SCr levels are associated with the occurrence and progression of stroke. However, no relevant studies have been conducted in AIS patients with impaired renal function. Our study conducted a prospective analysis of 1,932 patients (mean age: 66.7 ± 11.3 years) with AIS and abnormal renal function to investigate the causal relationship between SUA/SCr and stroke recurrence in patients with abnormal renal function.

Gong et al. utilized data from the Third China National Stroke Registry III and found that lower SUA/SCr values were associated with poor functional outcomes at 3 months and 1 year (31). The association between SUA/SCr and poor functional outcomes was non-linear, showing somewhat L-shaped associations. They also observed a trend suggesting a protective effect of SUA/SCr in patients with abnormal renal function (eGFR < 90 ml/min/1.73 m^2^), although the association did not reach statistical significance (31). Similarly, our results indicate a protective effect of SUA/SCr in patients with AIS and abnormal renal function. However, we found that lower values of SUA/SCr were associated with 1-year stroke recurrence, and this association was more significant in patients with eGFR < 60 ml/min/1.73 m^2^. SUA/SCr ratio and 1-year stroke recurrence were negatively and non-linearly associated. In addition to differences in research methodologies and outcomes, their study was based on national average data, whereas ours was based on regional data; consequently, our findings hold implications for regional prevention. Most previous studies have explored and analyzed the association between SUA levels and stroke outcomes, including poor prognosis and mortality. However, the association between SUA levels and stroke recurrence has been poorly reported. A meta-analysis of 23 articles, including 15,733 patients with AIS, showed that SUA has a linear dose-response relationship with outcomes in patients with AIS within a certain limit, with higher baseline SUA indicating better outcomes after AIS (9). Our observations are in line with this conclusion, indicating a protective effect of SUA/SCr on stroke outcomes.

Our study results suggest that among patients with impaired renal function (eGFR < 60 mL/min/1.73 m^2^), there may be a more pronounced non-linear negative correlation between SUA/SCr levels and the risk of stroke recurrence. The trend of changes across the Q1–Q4 groups showed significant statistical significance, which may indicate a threshold effect of SUA/SCr levels in this population. Specifically, when SUA/SCr levels are higher (Q4 group), the 1-year risk of stroke recurrence may be significantly lower compared to when SUA/SCr levels remain low (Q1–Q3 groups).

Relevant prior studies are limited. A review by Lee et al. (34) reported that baseline eGFR < 60 mL/min/1.73 m^2^ was independently associated with stroke events across various populations and study designs. Additionally, a study by Cui et al. (35) showed that lower eGFR levels were associated with the highest risk of cardiovascular disease, suggesting that renal function may act as a mediating factor in the relationship between cardiovascular risk factors. Both previous studies and our findings suggest that impaired renal function may also mediate the relationship between SUA/SCr levels and stroke recurrence in AIS patients.

Furthermore, by comparing the clinical characteristics of AIS patients with eGFR < 60 mL/min/1.73 m^2^ across SUA/SCr quartiles, we found that, compared to the Q1 group, patients in the Q4 group were younger, had lower blood urea nitrogen levels, and higher serum uric acid levels. Other clinical characteristics showed no statistically significant differences among the groups (Supplementary Table 2). This indicates that the relationship between SUA/SCr levels and stroke recurrence in patients with eGFR < 60 is likely influenced by these clinical characteristics. However, further validation in larger and more diverse populations is needed in future studies.

Previous studies have referred to patients with eGFR > 60 mL/min/1.73 m^2^ as having preserved renal function (29). Among these AIS patients, stroke risk decreased in the Q2 group compared to the Q1 group as SUA/SCr levels increased; however, the trend of changes across groups did not reach statistical significance. This finding suggests that variations in SUA/SCr levels may have a relatively minor impact on stroke recurrence risk in patients with preserved renal function. Although the results we obtained reflect an independent effect after adjusting for relevant confounding factors, the differences in clinical characteristics among the groups may also play a role. By comparing the clinical characteristics of AIS patients with eGFR > 60 across SUA/SCr quartiles, we observed that, compared to the Q1 group, patients in the Q2 group had lower FP and aspartate aminotransferase levels, as well as higher triglyceride and serum uric acid levels (Supplementary Table 3). These differences in clinical characteristics merit attention and warrant further investigation in subsequent studies.

Subgroup analysis revealed no significant interactions in patients with pneumonia, atrial fibrillation, or prior stroke. However, in patients without these conditions, the risk of 1-year stroke recurrence decreased significantly as SUA/SCr levels increased. Previous studies have indicated a positive correlation between SUA/SCr and the severity of dyspnea in patients with chronic obstructive pulmonary disease (COPD), suggesting its predictive value for COPD exacerbation and the severity of the disease (36). In addition, pneumonia has been linked to a higher risk of stroke recurrence (37), and these results support our findings. Interestingly, our subgroup analyses found no significant correlation between increased SUA/SCr values in patients with pneumonia and reduced stroke recurrence risk within 1 year. Similarly, previous studies have shown that AIS related to atrial fibrillation is associated with a high risk of recurrence (38). Prior stroke is also associated with an increased risk of recurrence (39). The results of these studies support our research findings. The association of SUA/SCr with the risk of stroke recurrence was less significant in patients with atrial fibrillation or prior stroke. In summary, for patients without pneumonia, atrial fibrillation, or prior stroke, it is essential to monitor SUA/SCr. Maintaining higher SUA/SCr values may potentially reduce the 1-year risk of stroke recurrence.

The mechanism linking high SUA/SCr with reduced stroke recurrence remains unclear. Previous studies suggest that one possible explanation is the direct antioxidant effect of SUA. As a powerful antioxidant, SUA contributes to approximately two-thirds of the antioxidant capacity in human plasma (40). In ischemic stroke, brain damage is primarily driven by the production of peroxynitrite and free radicals, which are highly reactive oxidant molecules (41). Peroxynitrite is mainly generated in the ischemic penumbra. Free radical levels gradually rise during cerebral ischemia, with a more pronounced increase occurring during reperfusion (42). SUA specifically inhibits peroxynitrite formation and acts as a scavenger of peroxynitrite (43). SUA effectively neutralizes free radicals generated from peroxynitrite decomposition (42). These findings suggest that SUA serves as an endogenous neuroprotective molecule, which may partly explain our results. In addition, our data analysis revealed that higher SUA/SCr levels in patients with AIS and abnormal renal function might be associated with younger age, less of diabetes mellitus and pneumonia, and lower initial stroke severity scores. This characteristic aligns with findings from previous studies, which indicate that stroke recurrence is more likely to occur in elderly patients, as well as in those with diabetes, pneumonia, or severe neurological impairment (37, 44–46). These findings highlight the importance of early attention to SUA/SCr levels, beyond routine interventions targeting comorbidities. Early recognition and timely intervention could significantly reduce the risk of 1-year stroke recurrence in patients with AIS and abnormal renal function. Furthermore, our study showed that higher SUA/SCr levels in these patients might also be associated with elevated triglyceride and aspartate aminotransferase levels. These characteristics suggest that higher SUA/SCr levels may be linked to increased dyslipidemia and liver dysfunction. This finding implies that abnormalities in lipid metabolism and liver function could influence the interaction between SUA/SCr levels and stroke recurrence in patients with AIS and abnormal renal function, warranting further in-depth research and analysis in the future. Our study has some limitations. Firstly, we did not record dynamic changes in the SUA/SCr, which may have affected the stroke outcomes during the follow-up period. Future studies should address this by monitoring these changes over time. Secondly, owing to the lack of data on patients undergoing mechanical thrombectomy, intravenous thrombolysis, or both, we could not analyze this subgroup. Finally, the selected hospitals were all tertiary hospitals in the urban area of Xi'an, and data from surrounding community hospitals were not collected, possibly introducing selection bias. In the future, we plan to expand the scope and amount of data for more in-depth research.

## 5 Conclusion

Low SUA/SCr is an independent predictor of 1-year stroke recurrence in AIS patients with abnormal renal function, particularly in those with eGFR < 60 mL/min/1.73 m^2^. This underscores the need for clinicians to closely monitor SUA/SCr levels in AIS patients with renal impairment.

## Data Availability

The original contributions presented in the study are included in the article/Supplementary material. Further inquiries can be directed to the corresponding author. Requests to access these datasets should be directed to Songdi Wu, wusongdi@gmail.com.
