# Supplementary material for: Serum uric acid/creatinine ratio and 1-year stroke recurrence in patient with acute ischemic stroke and abnormal renal function: results from the Xi'an stroke registry study of China
Source: Front Neurol. 2025 Feb 4;16:1496791. doi: 10.3389/fneur.2025.1496791 (PMC11832382; doi:10.3389/fneur.2025.1496791)
Supplement: Supplementary file 1 [file Table_1.docx]

**Supplementary Table 1 Comparative analysis of clinical characteristics of patients with 1-year follow up and lost to follow-up.**

| Variables | Total  (n=2148) | follow-up  (n=1932) | lost to follow-up (n=216) | P-value |
| --- | --- | --- | --- | --- |
| Age, Mean ± SD | 66.6 ± 11.3 | 66.7 ± 11.3 | 66.0 ± 11.4 | 0.416 |
| Sex, n (%) | |  |  | 0.22 |
| Male | 1411 (65.7) | 1261 (65.3) | 150 (69.4) |  |
| Female | 737 (34.3) | 671 (34.7) | 66 (30.6) |  |
| Medical insurance type, n (%) | |  |  | 0.117 |
| Urban employees’ medical insurance | 1034 (48.1) | 937 (48.5) | 97 (44.9) |  |
| New type rural cooperative medical system | 849 (39.5) | 756 (39.1) | 93 (43.1) |  |
| Commercial insurance | 10 (0.5) | 7 (0.4) | 3 (1.4) |  |
| Out-of-pocket medical | 255 (11.9) | 232 (12) | 23 (10.6) |  |
| Education level, n (%) | |  |  | 0.022 |
| Elementary or below | 1030 (48.0) | 932 (48.2) | 98 (45.4) |  |
| Middle school | 427 (19.9) | 369 (19.1) | 58 (26.9) |  |
| High school or above | 691 (32.2) | 631 (32.7) | 60 (27.8) |  |
| Smoking, n (%) | |  |  | 0.047 |
| Never smoking | 1172 (54.6) | 1058 (54.8) | 114 (52.8) |  |
| Smoking cessation | 466 (21.7) | 406 (21) | 60 (27.8) |  |
| Current smoking | 510 (23.7) | 468 (24.2) | 42 (19.4) |  |
| Alcohol consumption, n (%) | |  |  | 0.427 |
| No | 1638 (76.3) | 1478 (76.5) | 160 (74.1) |  |
| Yes | 510 (23.7) | 454 (23.5) | 56 (25.9) |  |
| Admission NIHSS score, (IQR) | 4.9 ± 5.2 | 5.0 ± 5.3 | 4.6 ± 4.4 | 0.257 |
| Total cholesterol (mmol/L) | 4.4 ± 1.1 | 4.4 ± 1.1 | 4.3 ± 1.0 | 0.72 |
| Triglycerides (mmol/L) | 1.6 ± 1.4 | 1.7 ± 1.4 | 1.5 ± 1.1 | 0.129 |
| HDL cholesterol (mmol/L) | 1.1 ± 0.3 | 1.1 ± 0.3 | 1.2 ± 0.3 | 0.137 |
| LDL cholesterol (mmol/L) | 2.6 ± 0.8 | 2.6 ± 0.8 | 2.6 ± 0.8 | 0.567 |
| Glycated hemoglobin (%) | 6.4 ± 1.6 | 6.4 ± 1.6 | 6.5 ± 1.6 | 0.742 |
| FPG (mmol/L) | 6.0 ± 2.4 | 6.0 ± 2.5 | 5.8 ± 2.3 | 0.154 |
| Alanine aminotransferase (U/L) | 23.2 ± 18.9 | 23.3 ± 19.1 | 22.5 ± 16.5 | 0.55 |
| Aspartate aminotransferase (U/L) | 24.7 ± 15.1 | 24.7 ± 15.2 | 24.5 ± 14.7 | 0.841 |
| Alkaline phosphatase (U/L) | 79.7 ± 28.6 | 79.8 ± 28.7 | 78.6 ± 27.9 | 0.547 |
| Homocysteine (µmol/L) | 22.1 ± 14.3 | 22.2 ± 14.5 | 21.8 ± 13.1 | 0.773 |
| Serum creatinine (µmol/L) | 76.7 ± 37.5 | 76.9 ± 38.7 | 75.3 ± 24.3 | 0.563 |
| eGFR (mL/min/1.73m^2^) | 72.5 ± 13.3 | 72.5 ± 13.3 | 71.9 ± 13.1 | 0.483 |
| Blood urea nitrogen (mmol/L) | 5.3 ± 2.0 | 5.2 ± 2.0 | 5.4 ± 2.1 | 0.254 |
| INR | 1.0 ± 0.2 | 1.0 ± 0.2 | 1.0 ± 0.1 | 0.348 |
| Serum uric acid (µmol/L) | 289.0 ± 98.3 | 288.2 ± 98.3 | 296.4 ± 97.9 | 0.246 |
| SUA/SCr | 4.0 ± 1.6 | 4.0 ± 1.5 | 4.2 ± 1.9 | 0.094 |
| White blood cell (×10^9^/L) | 7.0 ± 2.6 | 7.0 ± 2.6 | 6.7 ± 2.4 | 0.065 |
| Platelet count (×10^9^/L) | 187.2 ± 59.9 | 187.4 ± 60.3 | 184.7 ± 56.5 | 0.532 |
| BMI (kg/m ^2^) | 23.7 ± 3.3 | 23.7 ± 3.4 | 23.8 ± 2.6 | 0.723 |
| SBP on admission (mmHg) | 146.2 ± 21.7 | 146.6 ± 21.9 | 143.0 ± 19.7 | 0.019 |
| DBP on admission (mmHg) | 85.4 ± 12.4 | 85.5 ± 12.5 | 83.8 ± 11.4 | 0.053 |
| HR (beats/min) | 75.0 ± 10.9 | 75.0 ± 10.9 | 75.4 ± 10.5 | 0.563 |
| Hypertension, n (%) | |  |  | 0.755 |
| No | 607 (28.3) | 544 (28.2) | 63 (29.2) |  |
| Yes | 1541 (71.7) | 1388 (71.8) | 153 (70.8) |  |
| Diabetes mellitus, n (%) | |  |  | 0.454 |
| No | 1616 (75.2) | 1458 (75.5) | 158 (73.1) |  |
| Yes | 532 (24.8) | 474 (24.5) | 58 (26.9) |  |
| Atrial fibrillation, n (%) | |  |  | 0.048 |
| No | 1986 (92.5) | 1779 (92.1) | 207 (95.8) |  |
| Yes | 162 (7.5) | 153 (7.9) | 9 (4.2) |  |
| Prior stroke, n (%) |  |  |  | 0.623 |
| No | 1503 (70.0) | 1355 (70.1) | 148 (68.5) |  |
| Yes | 645 (30.0) | 577 (29.9) | 68 (31.5) |  |
| Pneumonia, n (%) |  |  |  | 0.169 |
| No | 2024 (94.2) | 1816 (94) | 208 (96.3) |  |
| Yes | 124 (5.8) | 116 (6) | 8 (3.7) |  |

Note: NIHSS, National Institutes of Health Stroke Scale; HLD, High-Density Lipoprotein; LDL, Low-Density Lipoprotein; FPG, Fasting Venous Plasma Glucose；eGFR, estimated Glomerular Filtration Rate; INR, international normalized ratio; BMI, Body Mass Index; SBP, Systolic Blood Pressure; DBP, Diastolic Blood Pressure; HR, Heart Rate.
