# Supplementary material for: Serum uric acid/creatinine ratio and 1-year stroke recurrence in patient with acute ischemic stroke and abnormal renal function: results from the Xi'an stroke registry study of China
Source: Front Neurol. 2025 Feb 4;16:1496791. doi: 10.3389/fneur.2025.1496791 (PMC11832382; doi:10.3389/fneur.2025.1496791)
Supplement: Supplementary file 2 [file Table_2.docx]

|  |
| --- |

**Supplementary Table 2 Analysis of Clinical Characteristics of AIS Patients with eGFR <60 mL/min/1.73m² by SUA/SCr Quartiles**

| Variables | SUA/SCr quartile | | | | P-value |
| --- | --- | --- | --- | --- | --- |
|  | Q1(128) | Q2(61) | Q3(33) | Q4(64) |  |
| Age (years) | 70.7 ± 10.9 | 74.5 ± 9.8 | 75.1 ± 8.9 | 69.7 ± 13.3 | 0.019 |
| Sex, n (%) |  |  |  |  | 0.222 |
| Male | 75 (58.6%) | 37 (60.7%) | 22 (66.7%) | 47 (73.4%) |  |
| Female | 53 (41.4%) | 24 (39.3%) | 11 (33.3%) | 17 (26.6%) |  |
| Smoking, n (%) |  |  |  |  | 0.503 |
| Never smoking | 85 (66.4%) | 39 (63.9%) | 21 (63.6%) | 33 (51.6%) |  |
| Smoking cessation | 22 (17.2%) | 13 (21.3%) | 8 (24.2%) | 16 (25.0%) |  |
| Current smoking | 21 (16.4%) | 9 (14.8%) | 4 (12.1%) | 15 (23.4%) |  |
| Alcohol consumption, n (%) |  |  |  |  | 0.829 |
| No | 109 (85.2%) | 50 (82.0%) | 26 (78.8%) | 53 (82.8%) |  |
| Yes | 19 (14.8%) | 11 (18.0%) | 7 (21.2%) | 11 (17.2%) |  |
| Hypertension, n (%) |  |  |  |  | 0.640 |
| No | 17 (13.3%) | 12 (19.7%) | 5 (15.2%) | 12 (18.8%) |  |
| Yes | 111 (86.7%) | 49 (80.3%) | 28 (84.8%) | 52 (81.2%) |  |
| Diabetes mellitus, n (%) |  |  |  |  | 0.669 |
| No | 84 (65.6%) | 44 (72.1%) | 24 (72.7%) | 41 (64.1%) |  |
| Yes | 44 (34.4%) | 17 (27.9%) | 9 (27.3%) | 23 (35.9%) |  |
| Atrial fibrillation, n (%) |  |  |  |  | 0.769 |
| No | 110 (85.9%) | 52 (85.2%) | 26 (78.8%) | 55 (85.9%) |  |
| Yes | 18 (14.1%) | 9 (14.8%) | 7 (21.2%) | 9 (14.1%) |  |
| Prior stroke, n (%) |  |  |  |  | 0.512 |
| No | 81 (63.3%) | 39 (63.9%) | 19 (57.6%) | 46 (71.9%) |  |
| Yes | 47 (36.7%) | 22 (36.1%) | 14 (42.4%) | 18 (28.1%) |  |
| BMI (kg/m^2^) | 23.5 ± 3.1 | 23.4 ± 3.1 | 23.3 ± 2.6 | 23.3 ± 4.6 | 0.978 |
| Admission NIHSS score, (IQR) | 4.0(3.0-8.0) | 4.0(3.0-8.0) | 4.0(3.0-10.0) | 4.0(3.0-8.2) | 0.589 |
| Totalcholesterol (mmol/L) | 4.4 ± 1.1 | 4.5 ± 1.3 | 4.4 ± 1.1 | 4.2 ± 1.4 | 0.536 |
| Triglycerides (mmol/L) | 1.7 ± 1.2 | 1.5 ± 1.0 | 1.4 ± 0.7 | 1.9 ± 2.5 | 0.376 |
| HDL cholesterol (mmol/L) | 1.1 ± 0.3 | 1.2 ± 0.4 | 1.2 ± 0.3 | 1.1 ± 0.4 | 0.379 |
| LDL cholesterol (mmol/L) | 2.6 ± 0.8 | 2.7 ± 1.0 | 2.6 ± 0.7 | 2.4 ± 0.7 | 0.144 |
| FPG (mmol/L) | 6.4 ± 3.1 | 5.8 ± 2.1 | 6.1 ± 2.0 | 6.4 ± 2.8 | 0.465 |
| Alanine aminotransferase(U/L) | 23.1 ± 34.1 | 19.0 ± 10.4 | 24.6 ± 30.8 | 25.8 ± 18.9 | 0.553 |
| Aspartate  aminotransferase(U/L) | 28.2 ± 33.4 | 23.0 ± 9.3 | 31.5 ± 37.3 | 28.0 ± 20.8 | 0.508 |
| Alkalinephosphatase (U/L) | 82.8 ± 29.6 | 78.6 ± 32.6 | 79.0 ± 27.4 | 81.9 ± 30.8 | 0.804 |
| Blood urea nitrogen (mmol/L) | 7.6 ± 3.1 | 6.5 ± 2.5 | 6.1 ± 2.1 | 5.1 ± 2.2 | <0.001 |
| Serum uric acid (µmol/L) | 278.0 ± 116.8 | 344.3 ± 127.1 | 363.6 ± 132.0 | 322.8 ± 114.1 | <0.001 |
| White blood cell (×10^9^/L) | 7.5 ± 3.5 | 7.4 ± 2.3 | 8.3 ± 3.1 | 7.2 ± 3.5 | 0.409 |
| Platelet count (×10^9^/L) | 183.3 ± 70.0 | 186.2 ± 63.2 | 185.6 ± 69.1 | 182.1 ± 57.6 | 0.984 |
