# Supplementary material for: Serum uric acid/creatinine ratio and 1-year stroke recurrence in patient with acute ischemic stroke and abnormal renal function: results from the Xi'an stroke registry study of China
Source: Front Neurol. 2025 Feb 4;16:1496791. doi: 10.3389/fneur.2025.1496791 (PMC11832382; doi:10.3389/fneur.2025.1496791)
Supplement: Supplementary file 3 [file Table_3.docx]

**Supplementary Table 3 Analysis of Clinical Characteristics of Patients with eGFR ≥60 mL/min/1.73m² by SUA/SCr Quartiles**

| Variables | SUA/SCr quartile | | | | P-value |
| --- | --- | --- | --- | --- | --- |
|  | Q1(355) | Q2(422) | Q3(50) | Q4(419) |  |
| Age (years) | 66.8 ± 10.1 | 67.1 ± 10.6 | 65.7 ± 11.5 | 63.7 ± 11.6 | <0.001 |
| Sex, n (%) |  |  |  |  | 0.111 |
| Male | 235 (66.2%) | 291 (69.0%) | 298 (66.2%) | 256 (61.1%) |  |
| Female | 120 (33.8%) | 131 (31.0%) | 152 (33.8%) | 163 (38.9%) |  |
| Smoking, n (%) |  |  |  |  | 0.686 |
| Never smoking | 198 (55.8%) | 221 (52.4%) | 231 (51.3%) | 230 (54.9%) |  |
| Smoking cessation | 69 (19.4%) | 99 (23.5%) | 95 (21.1%) | 84 (20.0%) |  |
| Current smoking | 88 (24.8%) | 102 (24.2%) | 124 (27.6%) | 105 (25.1%) |  |
| Alcohol consumption, n (%) |  |  |  |  | 0.356 |
| No | 278 (78.3%) | 322 (76.3%) | 332 (73.8%) | 308 (73.5%) |  |
| Yes | 77 (21.7%) | 100 (23.7%) | 118 (26.2%) | 111 (26.5%) |  |
| Hypertension, n (%) |  |  |  |  | 0.519 |
| No | 117 (33.0%) | 131 (31.0%) | 129 (28.7%) | 121 (28.9%) |  |
| Yes | 238 (67.0%) | 291 (69.0%) | 321 (71.3%) | 298 (71.1%) |  |
| Diabetes mellitus, n (%) |  |  |  |  | 0.135 |
| No | 258 (72.7%) | 323 (76.5%) | 358 (79.6%) | 326 (77.8%) |  |
| Yes | 97 (27.3%) | 99 (23.5%) | 92 (20.4%) | 93 (22.2%) |  |
| Atrial fibrillation, n (%) |  |  |  |  | 0.753 |
| No | 333 (93.8%) | 389 (92.2%) | 422 (93.8%) | 392 (93.6%) |  |
| Yes | 22 (6.2%) | 33 (7.8%) | 28 (6.2%) | 27 (6.4%) |  |
| Prior stroke, n (%) |  |  |  |  | 0.574 |
| No | 243 (68.5%) | 298 (70.6%) | 327 (72.7%) | 302 (72.1%) |  |
| Yes | 112 (31.5%) | 124 (29.4%) | 123 (27.3%) | 117 (27.9%) |  |
| BMI (kg/m^2^) | 23.7 ± 3.8 | 23.7 ± 3.1 | 23.6 ± 3.3 | 24.2 ± 3.3 | 0.025 |
| Admission NIHSS score, (IQR) | 4.0(3.0-8.0) | 4.0(3.0-8.0) | 4.0(3.0-8.0) | 4.0(3.0-8.0) | 0.104 |
| Totalcholesterol (mmol/L) | 4.3 ± 1.0 | 4.3 ± 1.1 | 4.4 ± 1.0 | 4.5 ± 1.1 | 0.294 |
| Triglycerides (mmol/L) | 1.5 ± 1.2 | 1.6 ± 1.2 | 1.7 ± 1.4 | 1.9 ± 1.6 | <0.001 |
| HDL cholesterol (mmol/L) | 1.1 ± 0.3 | 1.1 ± 0.3 | 1.1 ± 0.3 | 1.1 ± 0.3 | 0.584 |
| LDL cholesterol (mmol/L) | 2.6 ± 0.8 | 2.6 ± 0.8 | 2.6 ± 0.8 | 2.7 ± 0.8 | 0.248 |
| FPG (mmol/L) | 6.5 ± 2.9 | 5.9 ± 2.5 | 5.8 ± 2.1 | 5.9 ± 2.1 | <0.001 |
| Alanine aminotransferase(U/L) | 23.1 ± 19.2 | 21.1 ± 12.5 | 23.6 ± 20.3 | 25.6 ± 16.6 | 0.003 |
| Aspartate  aminotransferase(U/L) | 25.7 ± 15.7 | 22.7 ± 8.2 | 23.3 ± 9.8 | 25.3 ± 12.1 | <0.001 |
| Alkalinephosphatase (U/L) | 80.3 ± 30.1 | 78.0 ± 23.1 | 81.2 ± 26.9 | 78.9 ± 32.9 | 0.356 |
| Blood urea nitrogen (mmol/L) | 5.0 ± 1.6 | 5.1 ± 1.9 | 5.0 ± 1.5 | 4.9 ± 1.6 | 0.215 |
| Serum uric acid (µmol/L) | 185.5 ± 66.4 | 265.5 ± 55.1 | 302.7 ± 61.5 | 366.2 ± 84.2 | <0.001 |
| White blood cell (×10^9^/L) | 7.2 ± 3.0 | 6.8 ± 2.5 | 6.8 ± 2.3 | 7.0 ± 2.2 | 0.050 |
| Platelet count (×10^9^/L) | 183.2 ± 60.5 | 190.3 ± 63.2 | 188.3 ± 55.6 | 189.6 ± 58.2 | 0.361 |
